# Construction of a Secondary Enclosure for UVB Irradiation of Mice

**DOI:** 10.1016/j.xjidi.2022.100164

**Published:** 2022-09-29

**Authors:** Justin Choi, Zachary A. Bordeaux, Gabriella Braun, Cole Davis, Varsha Parthasarathy, Junwen Deng, Mathew T. Taylor, Anusha Kambala, Hannah Cornman, Olusola Oladipo, Martin P. Alphonse, Cameron E. West, Shawn G. Kwatra, Madan M. Kwatra

**Affiliations:** 1 Department of Dermatology, Johns Hopkins University School of Medicine, Baltimore, Maryland, USA; 2 Department of Anesthesiology, Duke University School of Medicine, Durham, North Carolina, USA; 3 Genzada Pharmaceuticals, Hutchinson, Kansas, USA; 4 Department of Oncology, Johns Hopkins University School of Medicine, Baltimore, Maryland, USA; 5 Department of Pharmacology & Cancer Biology, Duke University School of Medicine, Durham, North Carolina, USA

## Abstract

UV irradiation is commonly used in murine models of skin cancers. Despite the popularity of using UVB rays to model photocarcinogenesis in animals, there is a lack of standardization in the secondary enclosures used to administer radiation. An appraisal of the literature also shows a general lack of details regarding the materials and procedures utilized in the fabrication of such enclosures. We present in this study a detailed overview of the construction of a UVB exposure chamber that successfully induces lesions in hairless mice. A standardized protocol for producing a UVB enclosure may reduce methodological variation in future studies seeking to investigate photocarcinogenesis in animals.

## Introduction

UVB irradiation is a common method that has been used to reproduce cutaneous lesions in murine models of photodamage-induced skin cancer. Although UVB irradiation has allowed researchers to investigate the pathogenesis of photocarcinogenesis and validate novel therapies against pertinent dermatologic malignancies, there is a lack of standardization in the secondary enclosures and UV lighting systems used to conduct these studies (Table 1).

**Table 1:** Select Studies Using UVB Carcinogenesis

Mouse	Light Source	Enclosure Details/Dimensions	Reference
SKH-1	Philips TL-12/40W	Bulb 23‒26 cm above the cage	Baek et al., 2018
SKH-1	Philips TL-12/40W	None	Bertelsen et al., 2016
SKH-1	Oriel solar simulators	None	Chastkofsky et al., 2015
SKH-1	FS72T12-UVB-HO	None	Dinkova-Kostova et al., 2008
SKH-1	UV6 tubes	None	Erlendsson et al., 2016
SKH-1	Daavlin Research Irradiator	Premanufactured UV cabinet	Mintie et al., 2020
SKH-1	F72T12 100W/12 Phillips UVB Broadband TL	None	Phillips et al., 2013
SKH-1	Vilber Lourmat T-40.M	Customized cabinet with 28 cage capacity	Pillon et al., 2017
SKH-1	Philips TL-12/40W	None	Rebel et al., 2012, 2001
C57BL/6J	Sankyo Denki UVB lamp	None	Ho et al., 2020
C57BL/6	UV-LED chips	3D printed darkroom module	Lin et al., 2021
NMRI-HR-HR	TL12/20W	180 cm above the cage	Boiy et al., 2011
HRS/J	Philips TL-12/40W	1.3 × 0.43 × 0.45 m box bulb 15 cm above the cage	Carrara et al., 2019; Kremer et al., 2019
SENCAR *Ptk6‒/‒*	FB-UVXL-1000 UV crosslinker	Premanufactured UV cabinet	Chastkofsky et al., 2015
HR-1	Handheld UVM-57 lamp	None	Murata et al., 2021
HRM-2	IedaBoeki UVB lamp	None	Saba et al., 2020
ICR-Foxn/nu	Bio-sun illuminator system	Bulb 10 cm above the cage	Wang et al., 2019

Abbreviations: 3D, three-dimensional; LED, light emitting diode;

The systems found in the existing literature fall into one of two broad categories: exposure chambers fabricated by investigators (or prefabricated enclosures that have been secondarily co-opted for this purpose [Carrara et al., 2019; Kremer et al., 2019]) and, less commonly, manufactured UV cabinets. The cost of premanufactured UV cabinets may represent a barrier for investigators. Although fabricating a dedicated enclosure may be more cost effective and tailored for laboratory-specific needs, an appraisal of the literature shows minimal information and standardization across the UV exposure chambers used in different studies, particularly with regard to the dimensions or specific materials used in their construction. This is further complicated by the variation in the specific UVB source and exposure regimens used to induce lesions.

We present in this study the materials used and steps outlining the construction of a secondary UV exposure chamber as well as an exposure regimen that produces cutaneous lesions in hairless mice. A standardized protocol for developing a UV enclosure will aid future investigators in maximizing reproducibility and minimizing methodological variation.

## Results

### System functionality

**Figure 2:**
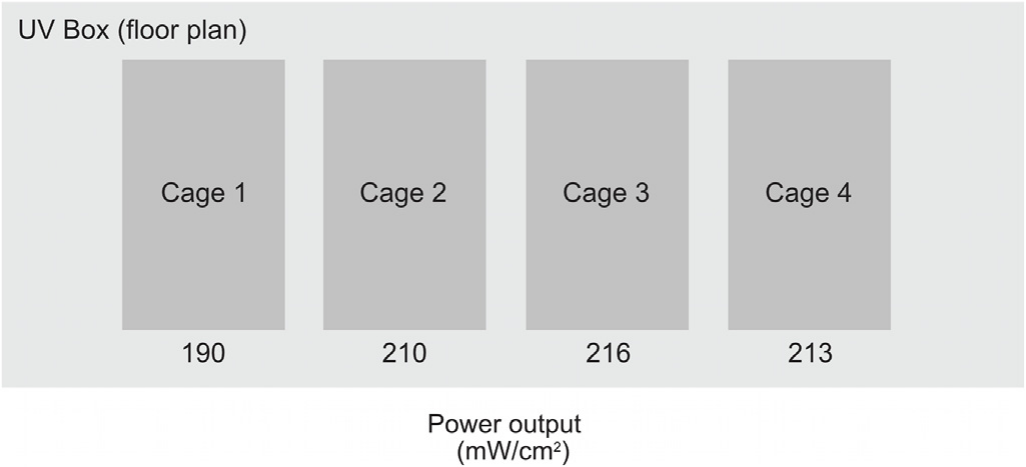
**Inconsistencies in power output along the length of the UVB bulb.** The floorplan of the box with representative power outputs noted under each cage position is shown, showing diminished power output localized to the position under the lamp corresponding to cage 1.

Given that the fluorescent bulb is 4 feet long, the length of the lamp is sufficient for irradiating four standard mice cages placed side by side (Figure 2), with cages being placed directly under the light source. Given that each cage can house up to five mice, the enclosure described in this study has the capacity to administer UVB radiation to up to 20 mice simultaneously. Because cages could not have their factory default lids during irradiation, a simple wire rack is effective in ensuring that mice do not climb out of the cages when placed in the enclosure while also allowing sufficient penetration of UVB into the cages. System maintenance was not required for the chamber beyond routine disinfection.

### Lamp characteristics

The time required to reach a stable power output during warm-up is approximately 3 minutes, after which the experimental irradiation could take place. The average power output was approximately 180 *μ*W/cm^2^, equating to 278 seconds of exposure per irradiation period. Although minor week-to-week fluctuations were noted in power output, a global decrease in power was not noted after 6 months of bulb usage. Variation in power output was also observed along the length of the lamp, showing an approximately 10% decrease at the distal end of the bulb unilaterally (Figure 2). To ensure equal UVB dosage, the positions in which cages were placed during UVB administration were systematically rotated.

### Lesion formation

**Figure 3:**
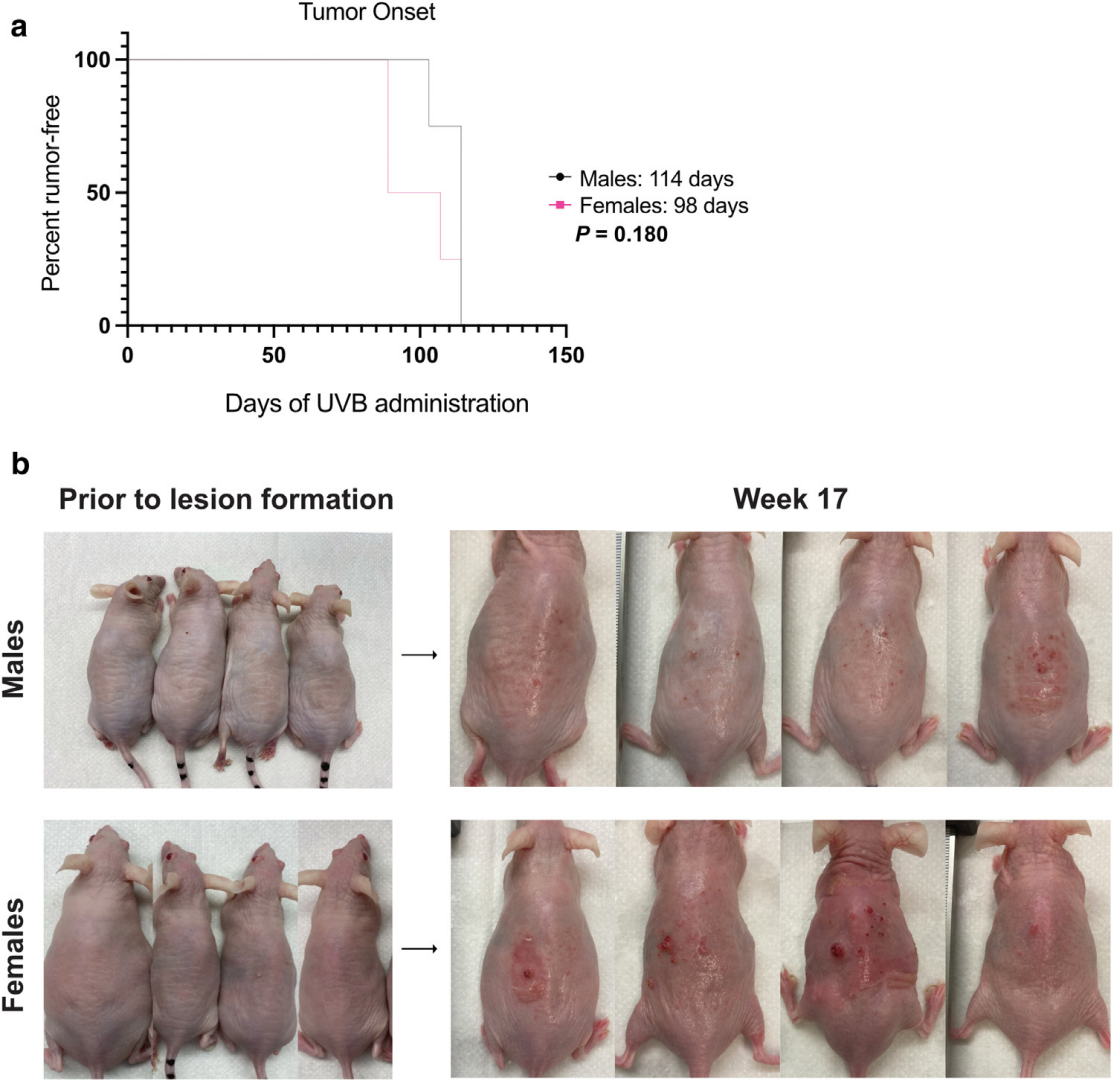
**The UVB enclosure and lighting system induce lesion formation in SKH-1 mice.** (**a**) Kaplan‒Meier analysis of tumor-free mice showing that lesions appeared after approximately 13 weeks of UVB irradiation, and all mice developed lesions by week 16, with no significant difference between genders. (**b**) Representative images of mice before lesion formation and after 17 weeks of UVB exposure.

Following a regimen that irradiated mice with 500 J/m^2^ of UVB five times per week, the time to the appearance of the first lesion was approximately 13 weeks, with all mice showing lesions by approximately 16 weeks (Figure 3a and b).

## Discussion and Potential Applications

The UV system described in this paper was built with a raw material cost of 450 United States dollars and provides a practical method for fabricating an enclosure containing a UVB source that induces lesion formation in hairless SKH-1 mice after approximately 13 weeks. Explicit requirements established by Institutional Animal Care and Use Committee at our institution included construction with nonporous materials (for disinfection), a locking mechanism (to prevent mouse escape), ventilation holes, and an alternative lid that both prevented mouse escape and allowed sufficient UVB penetration into the cage. Proper care should be taken to ensure that the fenestrations of a given alternative lid are large enough for UVB to penetrate. Future efforts should be aimed toward characterizing the use of transparent barriers with a minimal reflective capacity to maximize UVB administration. Knowledge of the basic requirements of the Institutional Animal Care and Use Committee at our institution may allow other investigators to proactively fulfill similar requirements established by parallel ethical committees when constructing similar enclosures, thus expediting approval for animal studies.

Our UV exposure protocol was adapted from investigators using a fixed irradiation time throughout the duration of the experiment (Baek et al., 2018); however, potential temporal degradation of power output necessitated regular internal recalibration of exposure time. Although no power degradation was noted in our UVB source after 6 months of use, gradual temporal degradation reported in previous studies reiterates the importance of routinely checking output and adjusting exposure times (Pillon et al., 2017). Our study also showed variations in power output along the length of the lamp. An appraisal of the literature did not identify any study that accounted for this factor when irradiating mice, whereas only one study noted that consistent power output was achieved when using UVB light-emitting diode chips (Lin et al., 2021), given that the energy is equally distributed among individual diodes rather than a single fluorescent tube. Thus, a potential method for further optimizing this enclosure may lie in the use of UV light-emitting diode chips as opposed to a fluorescent bulb to maximize consistency.

These factors are of particular significance because insufficient irradiation may prolong the time to lesion formation, whereas inconsistent UVB dosage may lead to differences in time to lesion formation as well as lesion severity among mice. To account for this, cage positions were rotated on a daily basis to ensure that mice received equitable radiation dosages over the experimental period. Although this may be an individual bulb-specific phenomenon, our experiences suggest that investigators utilizing fluorescent UV tubes should measure power output along the length of the lamp to ensure that appropriate countermeasures are taken to equalize UV administration among cages and prevent undue methodological variation.

With the anticipated increase in the prevalence of sun-induced dermatologic malignancies, UV irradiation systems will continue to grow in relevance in the realm of skin cancer research and drug development. A standardized protocol for constructing a cost-effective enclosure may help to reduce variation and methodological errors in future studies seeking to investigate photocarcinogenesis in murine models.

## Materials and Methods

### Materials and construction of the enclosure

**Figure 1:**
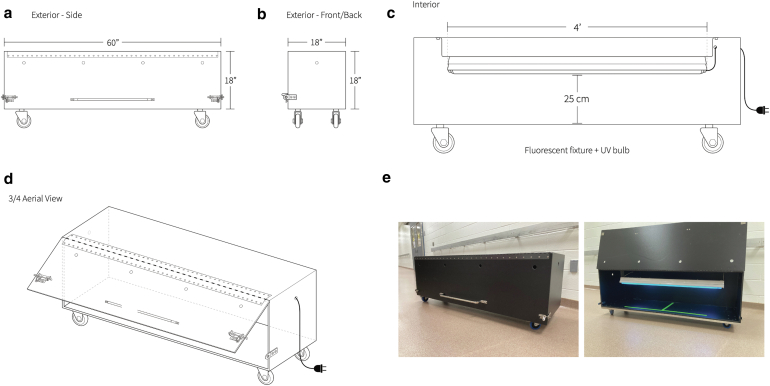
**Secondary UVB exposure chamber.** Blueprint schematic of the enclosure from the (**a**) exterior side view, (**b**) exterior frontal view, (**c**) interior side view, and (**d**) three quarter aerial view. (**e**) Representative images of the enclosure. Illustrations were by Caroline Choi.

All materials used to construct the enclosure are listed in Table 2 and were purchased from a hardware store at an approximate cost of 450 United States dollars. The UV exposure chamber was fabricated with a black polyvinyl chloride board with the following dimensions: 60 × 18 × 18 inches. The decision to utilize polyvinyl chloride board in the construction of the enclosure was informed by the observation that certain nonporous materials are subject to degradation when exposed to commercial disinfectants. All components were secured using stainless steel screws, whereas the box joints were reinforced with plastic cement. Pipe cement was used to seal the joint seams of the box’s interior, and ventilation holes were drilled into all four walls to permit airflow. An additional hole was fitted with a sealing grommet and served as an outlet feeding the wire from the light fixture to the exterior. A single TL 40W12 RS SLV/25 fluorescent tube (Phillips, Amsterdam, Netherlands) was mounted on a Lithonia T12 2-light fixture (Lithonia Lighting, Atlanta, GA) 25 cm above the floor of the enclosure (Baek et al., 2018). S-brackets were used to mount the fixture to the ceiling of the chamber’s interior. The superior edge of one 60 × 18 inch wall was secured to a piano hinge to serve as a door, with a handlebar and packlockable latches on both sides of the door to enable locking, thus preventing mouse escape during experiments. Four casters were secured to the underside of the enclosure for mobility, two of which were locking (Figure 1a‒d).

**Table 2:** Materials Used in the Construction of the Enclosure

Item	Quantity	Purpose
½” thick polyvinyl chloride board	User defined	Box structure
Philips TL 40W12 RS SLV/25 bulb	1	UVB source
Lithonia T12 2-light fixture	1	Bulb fixture
S-bracket	2	Mount fixture to the box
Handlebar	1	Handle for opening door
18-8 stainless steel screws	User defined	Securing all components
Lift-and-drop padlockable latch	2	Door lock
Pipe cement	User defined	Seal internal seams
Cement for plastic	User defined	Reinforce joints
Sealing grommet	1	Feed wire from interior to exterior
Caster (lockable)	4	Mobility
Piano hinge	1	Door functionality

### UV exposure regimen

The UV exposure regimen was adopted from the protocol outlined by Baek et al. (2018). Eight male (n = 4) and female (n = 4) SKH-1 mice (Charles River Laboratories, Wilmington, MA) aged between 6 and 8 weeks were used for this study. Four mice were housed per cage, and their skins were monitored daily for signs of injury. Before UV exposure, the bulb was allowed to warm up until it reached its maximum power output, measured using a PM100D power meter and S120VC power sensor (Thor Labs, Newton, NJ) (Baek et al., 2018). Power output was calculated by measuring the power sensor in six different locations within each cage and across all cage positions within the enclosure. The final readout was calculated as the average of all power measurements and was used to determine exposure time. Exposure time was internally recalibrated on a weekly basis to account for temporal power degradation (Pillon et al., 2017). Mice were irradiated with 500 J/m^2^ of UVB (the minimum erythemal dose of these lamps identified in several independent studies) five times per week for 14 weeks (Baek et al., 2018; Rebel et al., 2012, 2001; Voskamp et al., 2012). Of note, strains of hairy mice, such as C57BL/6 and BALB/c, are often shaven and used for models of UV-induced carcinogenesis. For these mice, the minimum erythemal dose has been reported to be higher than in SKH-1 mice, ranging from 1,200 to 1,900 J/m^2^ for C57BL/6 (Memari et al., 2019; Skobowiat and Slominski, 2015; Toriyama et al., 2021) and 1,500 to 2,250 J/m^2^ for BALB/c (Jeevan and Kripke, 1990; Toriyama et al., 2021). Before UV exposure, the factory lids and stainless-steel cage covers were removed from the cages, and a flat wire grid rack was placed on top of the open cages to prevent mice from escaping during irradiation. Of note, power output was measured under these racks as described previously to best mimic the conditions under which mice were irradiated. The positions in which cages were placed for each irradiation period were systematically rotated to ensure equal radiation administration.

### Ethical approval

The chamber was compliant with the Institutional Animal Care and Use Committee at Duke University School of Medicine (Baltimore, MD) (protocol number A155-20-07). Mice were monitored daily for signs of deteriorating health and weighed once per week. Humane endpoints included signs of discomfort, distress or pain, reduced mobility, inactivity, abnormal posture, lack of grooming, sudden weight loss exceeding 20%, lesion ulceration or necrosis, and lesion size exceeding 10 mm in diameter (Workman et al., 2010). Mice meeting these endpoint criteria were killed.

### Data availabilty statement

No large dataset was generated or analyzed for this study.

## ORCIDs

Justin Choi: http://orcid.org/0000-0002-8388-6876

Zachary A. Bordeaux: http://orcid.org/0000-0002-8833-6080

Gabriella Braun: https://orcid.org/0000-0003-4496-3696

Cole Davis: https://orcid.org/0000-0003-0253-8720

Varsha Parthasarathy: http://orcid.org/0000-0002-1422-772X

Junwen Deng: http://orcid.org/0000-0001-8393-8915

Matthew T. Taylor: http://orcid.org/0000-0001-7192-7936

Anusha Kambala: http://orcid.org/0000-0002-0350-8622

Hannah Cornman: http://orcid.org/0000-0003-1462-2479

Olusola Oladipo: http://orcid.org/0000-0001-9498-1492

Martin P. Alphonse: http://orcid.org/0000-0003-3447-1284

Cameron E. West: http://orcid.org/0000-0001-9673-9165

Shawn G. Kwatra: http://orcid.org/0000-0003-3736-1515

Madan M. Kwatra: http://orcid.org/0000-0002-6547-8852

## Conflict of Interest

SGK is an advisory board member/consultant for Abbvie, Celldex Therapeutics, Galderma, Incyte, Pfizer, Regeneron Pharmaceuticals, Kiniksa Pharmaceuticals, and Genzada Pharmaceuticals and has received grant funding from Galderma, Pfizer, and Kiniksa Pharmaceuticals. CEW is an officer and member of the Board of Directors at Genzada Pharmaceuticals.
